# Dose–Response Relationships Between Polypharmacy and All-Cause and Cause-Specific Mortality Among Older People

**DOI:** 10.1093/gerona/glab155

**Published:** 2021-06-03

**Authors:** Yun-Ting Huang, Andrew Steptoe, Li Wei, Paola Zaninotto

**Affiliations:** Department of Epidemiology and Public Health, University College London, UK; Department of Behavioural Science and Health, University College London, UK; School of Pharmacy, University College London, UK; Department of Epidemiology and Public Health, University College London, UK

**Keywords:** All-cause mortality, Cardiovascular disease mortality, Epidemiology, Heightened polypharmacy, Polypharmacy

## Abstract

**Background:**

Although medicines are prescribed based on clinical guidelines and expected to benefit patients, both positive and negative health outcomes have been reported associated with polypharmacy. Mortality is the main outcome, and information on cause-specific mortality is scarce. Hence, we investigated the association between different levels of polypharmacy and all-cause and cause-specific mortality among older adults.

**Method:**

The English Longitudinal Study of Ageing is a nationally representative study of people aged 50+. From 2012/2013, 6 295 individuals were followed up to April 2018 for all-cause and cause-specific mortality. Polypharmacy was defined as taking 5–9 long-term medications daily and heightened polypharmacy as 10+ medications. Cox proportional hazards regression and competing-risks regression were used to examine associations between polypharmacy and all-cause and cause-specific mortality, respectively.

**Results:**

Over a 6-year follow-up period, both polypharmacy (19.3%) and heightened polypharmacy (2.4%) were related to all-cause mortality, with hazard ratios of 1.51 (95% CI: 1.05–2.16) and 2.29 (95% CI: 1.40–3.75) respectively, compared with no medications, independently of demographic factors, serious illnesses and long-term conditions, cognitive function, and depression. Polypharmacy and heightened polypharmacy also showed 2.45 (95% CI: 1.13–5.29) and 3.67 (95% CI: 1.43–9.46) times higher risk of cardiovascular disease deaths, respectively. Cancer mortality was only related to heightened polypharmacy.

**Conclusion:**

Structured medication reviews are currently advised for heightened polypharmacy, but our results suggest that greater attention to polypharmacy in general for older people may reduce adverse effects and improve older adults’ health.

Polypharmacy is a legitimate response to multimorbidity, defined as the coexistence of 2 or more chronic conditions by the World Health Organization (1). Polypharmacy and multimorbidity are highly correlated and both of them are prevalent among older adults (1,2). Although there is no agreed definition of polypharmacy, the most common cutoff point described in the literature is 5, with 10 or more medications used to define a higher level of polypharmacy (3). Beyond the numerical definition, a concept of appropriate or problematic polypharmacy has been advocated by the National Institute for Health and Care Excellence (NICE) (4) and National Health Service (NHS) England (5). Some tools have also been developed to identify potentially inappropriate prescription combinations, such as Beers (6) and STOPP (7) criteria, and can help evaluate the appropriateness of polypharmacy. Nevertheless, the assessment of polypharmacy must be personalized and is often limited by data availability in population-based studies.

Although medicines are prescribed based on clinical guidelines and expected benefit to patients, not only positive but also negative health outcomes have been reported associated with polypharmacy (8,9). Several negative outcomes—falls, adverse drug events, hospitalization, mortality, functional decline, and cognitive impairment—have been studied widely in community-dwelling older adults (9). The literature on polypharmacy and mortality focuses on all-cause mortality, and information on cause-specific mortality is scarce. A meta-analysis (10) showed polypharmacy is associated with a higher risk of all-cause mortality, regardless of cutoff values of polypharmacy. Of the studies in the meta-analysis, many had short follow-ups; those with follow-ups of 5 years or more were based on selective nonrepresentative populations (10), making generalizability of the results difficult.

Furthermore, little is known about whether polypharmacy correlates with specific causes of death, such as cardiovascular disease (CVD), cancer, respiratory disease, and other causes. In clinical practice, current interventions in medication use target people with heightened polypharmacy rather than those with polypharmacy (5,11,12). Therefore, this study aimed to investigate the association between different levels of polypharmacy and all-cause and cause-specific mortality in a nationally representative sample of community-dwelling older adults in England.

## Methodology

### Study Population

Data came from wave 6 (2012–2013) of the English Longitudinal Study of Ageing (ELSA), a nationally representative study of adults in England age 50 and older living in private households (13). Data collection is carried out using computer-assisted interviews every 2 years, and home visits from a study nurse every 4 years in which blood samples and other health-related measurements are taken (14,15). At wave 6, a total of 9 169 interviews with core members were conducted. Of these, 7 730 participants were visited by a study nurse who recorded information on all medications. We excluded participants who had been diagnosed having cancer (*n* = 480), who had died within 1 year of follow-up (*n* = 82), and those without complete information on all variables (*n* = 905), so 6 295 participants were finally included in the study.

### Polypharmacy

Polypharmacy was defined as taking 5–9 long-term medications daily; taking 10 or more medications was defined as heightened polypharmacy. Heightened polypharmacy was employed instead of hyperpolypharmacy or excessive polypharmacy in order to avoid potentially negative implications from the terminology. Long-term medications were either drugs for chronic conditions such as cardiovascular and antihyperglycemic agents, or drugs for chronic symptoms such as sedatives for insomnia and opioid derivatives for pain relief. Over-the-counter (OTC) drugs used for chronic conditions were also included in this study, for example, calcium supplement for bone disease. Each distinct pharmacological agent was treated as an individual drug, so distinguishable combination drugs were counted based on the number of active ingredients.

### Mortality Data

Study participants were linked to the National Health Service’s Central Registry which provides vital status data. For each deceased participant, the month and year of death were recorded up to the end of follow-up (April 2018). Also, data regarding causes of death were provided for broad classifications of disease according to the International Classification of Diseases. These classifications include cancer (codes C00–C97), CVD (codes I00–I99), diseases of the respiratory system (codes J00–J99), and other remaining causes. For participants with no record of an event, the data were censored at the end of May 2018.

### Potential Confounders

#### Sociodemographic characteristics

A continuous variable for age was employed. Binary variables were gender (males and females) and cohabiting status (living, or not, with a partner). Wealth was used as the measure of economic resources, since it is more consistently associated with health outcomes at older ages than income (16). Wealth was computed from detailed assessments of housing wealth, savings, investments, and possessions net of debt (17,18) and was categorized into quintiles.

#### Health factors

Long-term conditions in ELSA Wave 6 were derived from either self-reported diagnoses or specific treatments. The self-reported diagnoses were also verified by medication information where it was possible. Six long-term conditions—diabetes mellitus, coronary heart disease (CHD), stroke, lung disease (including asthma), Parkinson’s disease, and Alzheimer’s disease and dementia—were included as individual covariates. The remaining chronic conditions—hypertension, other heart problems, hyperlipidemia, arthritis, bone disease, psychiatric conditions, eye disease, gout/hyperuricemia, epilepsy, and inflammatory bowel disease—were included in the models as an illness count for adjustment. Functional impairment was defined as self-reporting difficulty in either activities of daily living (ADLs) or instrumental ADLs (19,20). Mobility difficulty was defined as having difficulty in 10 movements of arms or lower limbs, such as walking 100 yards and picking up 5p coin from table (20). Obesity was derived from body mass index (BMI) and waist circumference, and categorized into “normal BMI and waist circumference,” “high BMI and waist circumference,” and “either high BMI or waist circumference.” The cutoff value of BMI was 30, and of waist circumference were 102 cm in males and 88 cm in females. Smoking status (ie, whether a current smoker or not) was also investigated. Sleep duration was categorized as binary, 7–9 hours versus less than 7 hours, or over 9 hours (21,22). Low physical activity was defined by self-report as not engaging in vigorous-/moderate-intensity activities at least once a week (20,23). Cognitive function was assessed by immediate and delayed recall memory tests, and scores ranged from 0 to 20 (24). People who self-reported 4 or more scores of the 8-item version of the Centre for Epidemiological Studies Depression Scale were classified as having significant depressive symptoms (25).

### Statistical Analysis

The association between polypharmacy and all-cause mortality was assessed by Cox proportional hazards regression, and hazard ratios (HRs) were reported. First, we estimated the age- and sex-adjusted model and then assessed the contribution of each set of factors separately. Lastly, the fully adjusted model was presented. The trend of HRs was tested by the likelihood ratio test.

Competing-risks regression based on Fine and Gray’s proportional subhazards model (26) was used to analyze cause-specific mortality, and subdistribution hazard ratios (SHRs) were reported. This method takes account of competing events that prevent the event of interest from occurring; for example, participants who died from CVD cannot die of other diseases. The proportionality of hazards and subhazards was tested by using Schoenfeld residuals (27,28) and no violation of assumptions was observed. Statistical analyses were conducted using Stata (version 15.1; StataCorp LP, College Station, TX).

#### Sensitivity analysis

Several sensitivity analyses were performed to ensure the robustness of main findings when adding specifically problematic drug–disease interactions (Supplementary Table S1), alcohol consumption (with reduced sample size), and an indicator of people who took medications but did not report relevant diagnoses to the main model. Since health status and death are strongly correlated, we also performed analyses with different adjustments of health status. Multimorbidity was used to replace long-term conditions, and all chronic conditions were adjusted individually rather than using an illness count. Lastly, all analyses were repeated when we treated taking 1–4 medications as the reference instead of no medications.

## Results

Of 6 295 participants, 1 844 (29.3%) did not take long-term medications, 3 088 (49.1%) took 1–4 medications a day, 1 214 (19.3%) took 5–9 medications (polypharmacy), and 149 (2.4%) took 10 or more medications (heightened polypharmacy). The cohort characteristics are summarized in Table 1. People classified into the polypharmacy and heightened polypharmacy categories tended to be older, be poorer, live without a partner, have more chronic conditions (particularly diabetes, CHD, stroke, lung disease, Parkinson’s disease, and Alzheimer’s disease and dementia, along with the number of the remaining conditions), report functional impairment and mobility difficulty, be obese, smoke currently, sleep inadequately, report low physical activity, have worse cognitive performance, and have significant depressive symptoms. Taking a greater number of drugs was also related to more all-cause and cause-specific deaths (Table 1).

**Table 1.** Baseline Characteristics and Mortality, According to the Number of Concurrent Drugs, ELSA 2012–2018

Variables^a^	None % (*n* = 1 844)	1–4 Drugs % (*n* = 3 088)	5–9 Drugs^b^ % (*n* = 1 214)	10+ Drugs^b^ % (*n* = 149)
Age (y), mean (*SD*)	62.9 (7.9)	67.8 (8.8)	71.9 (8.7)	71.8 (8.5)
Gender				
Men	47.1 (869)	42.3 (1 305)	45.3 (550)	41.6 (62)
Women	52.9 (975)	57.7 (1 783)	54.7 (664)	58.4 (87)
Total wealth				
1 (lowest)	15.1 (279)	18.1 (559)	28.2 (343)	33.6 (50)
2	16.1 (296)	20.2 (625)	23.2 (281)	21.5 (32)
3	19.9 (367)	20.4 (630)	19.4 (236)	22.1 (33)
4	23.3 (429)	20.2 (624)	18.2 (221)	12.1 (18)
5 (highest)	25.6 (473)	21.1 (650)	11.0 (133)	10.7 (16)
Live with a partner	75.2 (1 387)	71.3 (2 201)	63.3 (768)	56.4 (84)
Diabetes mellitus	1.7 (32)	9.8 (302)	33.2 (403)	49.0 (73)
CHD	0.6 (11)	5.1 (156)	26.8 (325)	48.3 (72)
Stroke	0.3 (6)	3.3 (102)	11.9 (144)	14.8 (22)
Lung disease (including asthma)	3.7 (69)	16.5 (510)	28.4 (345)	53.0 (79)
Parkinson’s disease	0.0 (0)	0.8 (25)	1.7 (21)	1.3 (2)
Alzheimer’s disease and dementia	0.2 (3)	0.5 (16)	1.9 (23)	2.7 (4)
Number of conditions^c^ median (IQR)	1.0 (1.0)	2.0 (2.0)	3.0 (2.0)	4.0 (2.0)
Functional impairment^d^	7.3 (135)	17.0 (524)	38.1 (463)	58.4 (87)
Mobility difficulty^e^	30.6 (564)	50.9 (1 571)	944 (77.8)	94.0 (140)
Obesity				
High BMI and waist circumference	20.0 (368)	28.2 (872)	41.9 (509)	53.0 (79)
Either high BMI or waist circumference	18.8 (346)	26.1 (806)	26.2 (318)	24.8 (37)
Current smoker	11.9 (219)	8.7 (269)	12.3 (149)	18.1 (27)
Sleep duration				
7–9 h	63.8 (1 177)	60.6 (1 873)	55.3 (671)	45.6 (68)
<7 or 9+ h	36.2 (667)	39.4 (1 215)	44.7 (543)	54.4 (81)
Low physical activity	8.8 (162)	17.3 (534)	35.8 (434)	63.1 (94)
Cognitive function, mean (*SD*)	11.9 (3.2)	11.0 (3.4)	9.8 (3.5)	8.7 (3.7)
Depressive symptoms: 4+	6.8 (126)	10.0 (309)	17.1 (207)	33.6 (50)
Mortality^f^				
All-cause death	3.1 (57)	6.6 (205)	16.1 (196)	27.5 (41)
Cause-specific deaths				
CVD	0.7 (13)	1.7 (51)	6.7 (81)	10.7 (16)
Cancer	1.4 (26)	2.4 (74)	4.0 (48)	8.1 (12)
Respiratory disease	0.4 (7)	0.8 (26)	2.4 (29)	5.4 (8)
Other cause	0.6 (11)	1.8 (54)	3.1 (38)	3.4 (5)

*Notes*: BMI = body mass index; CHD = coronary heart disease; CVD = cardiovascular disease; ELSA = English Longitudinal Study of Ageing; IQR = interquartile range.

^a^All variables had significantly different proportions among the 4 groups. ^b^Polypharmacy refers to taking 5–9 drugs, and heightened polypharmacy refers to taking ≥10 drugs. ^c^The rest of other conditions, not including diabetes mellitus, CHD, lung disease, Parkinson’s disease, and Alzheimer’s disease and dementia. ^d^Defined as any difficulty in either activities of daily living (ADLs) or instrumental ADLs. ^e^Defined as any difficulty in movements of arms or lower limbs. ^f^Data were collected before May 2018.

Table 2 shows the results of the association between the number of concurrent drugs and all-cause mortality from the Cox proportional hazards regressions. Concurrent use of 1–4 medications was not related to increased risk of death, whereas polypharmacy (HR = 1.51, 95% confidence interval [CI] = 1.05, 2.16) and heightened polypharmacy (HR = 2.29, 95% CI = 1.40, 3.75) showed a higher risk of death compared with no medication in the fully adjusted model. The linear trend further supported the dose–response relationship between polypharmacy and all-cause mortality. Statistical adjustment for long-term conditions led to the greatest attenuation of the hazards of polypharmacy (2.10–1.49) and heightened polypharmacy (4.22–2.51) on all-cause mortality, followed by adjustment for disability (functional impairment and mobility difficulty) and lifestyle factors (obesity, smoking status, sleep duration, and physical activity). Other factors—wealth and cohabitation, cognitive function, and depressive symptoms—also attenuated the associations with polypharmacy, but their impact was relatively small.

**Table 2.** Associations Between the Number of Concurrent Drugs and All-Cause Mortality, England 2012–2018

	None	1–4 Drugs		5–9 Drugs^a^		10+ Drugs^a^		
*N* = 6 295 (499 deaths)	HR	HR^a^ (95% CIs)	*p*	HR^a^ (95% CIs)	*p*	HR^a^ (95% CIs)	*p*	Trend^b^
Age and gender (basic model)	1.00 (ref.)	1.20 (0.89, 1.61)	.228	2.10 (1.55, 2.84)	**<.001**	4.22 (2.82, 6.33)	**<.001**	
Basic model + wealth and cohabitation	1.00 (ref.)	1.17 (0.86, 1.57)	.315	1.98 (1.46, 2.69)	**<.001**	3.93 (2.61, 5.91)	**<.001**	
Basic model + chronic conditions^c^	1.00 (ref.)	1.05 (0.77, 1.44)	.753	1.49 (1.04, 2.13)	**.031**	2.51 (1.54, 4.09)	**<.001**	
Basic model + disability^d^	1.00 (ref.)	1.14 (0.85, 1.54)	.386	1.85 (1.36, 2.51)	**<.001**	3.50 (2.31, 5.30)	**<.001**	
Basic model + lifestyle factors^e^	1.00 (ref.)	1.20 (0.89, 1.62)	.222	1.95 (1.43, 2.66)	**<.001**	3.58 (2.36, 5.45)	**<.001**	
Basic model + cognitive function	1.00 (ref.)	1.17 (0.87, 1.58)	.289	2.02 (1.49, 2.73)	**<.001**	3.81 (2.54, 5.72)	**<.001**	
Basic model + depressive symptoms	1.00 (ref.)	1.18 (0.88, 1.59)	.273	2.02 (1.49, 2.73)	**<.001**	3.97 (2.64, 5.96)	**<.001**	
All covariates (main model)	1.00 (ref.)	1.09 (0.80, 1.48)	.603	1.51 (1.05, 2.16)	**.026**	2.29 (1.40, 3.75)	**.001**	Linear

*Notes*: HR = hazard ratio.

^a^Polypharmacy refers to taking 5–9 drugs, and heightened polypharmacy refers to taking ≥10 drugs. ^b^Likelihood ratio test was used to test the trend of HRs, and *p* >.05 indicated that the trend was linear. ^c^Includes 6 chronic conditions (diabetes, coronary heart disease, stroke, lung disease [including asthma], Parkinson’s disease, and Alzheimer’s disease and dementia) and an illness count of the rest of the conditions. ^d^Includes functional impairment and mobility difficulty. ^e^Includes obesity and health behaviors: smoking status, sleep duration, and physical activity.

In addition to polypharmacy and heightened polypharmacy, factors significantly associated with a higher risk of death were older age, having diabetes, CHD, and lung disease, being a current smoker, and reporting low physical activity (Supplementary Table S2). By contrast, several factors linked to a lower risk of death, including being women, living with a partner, being obese (either or both high BMI and waist circumference), and showing better cognitive function.

**Figure 1.**
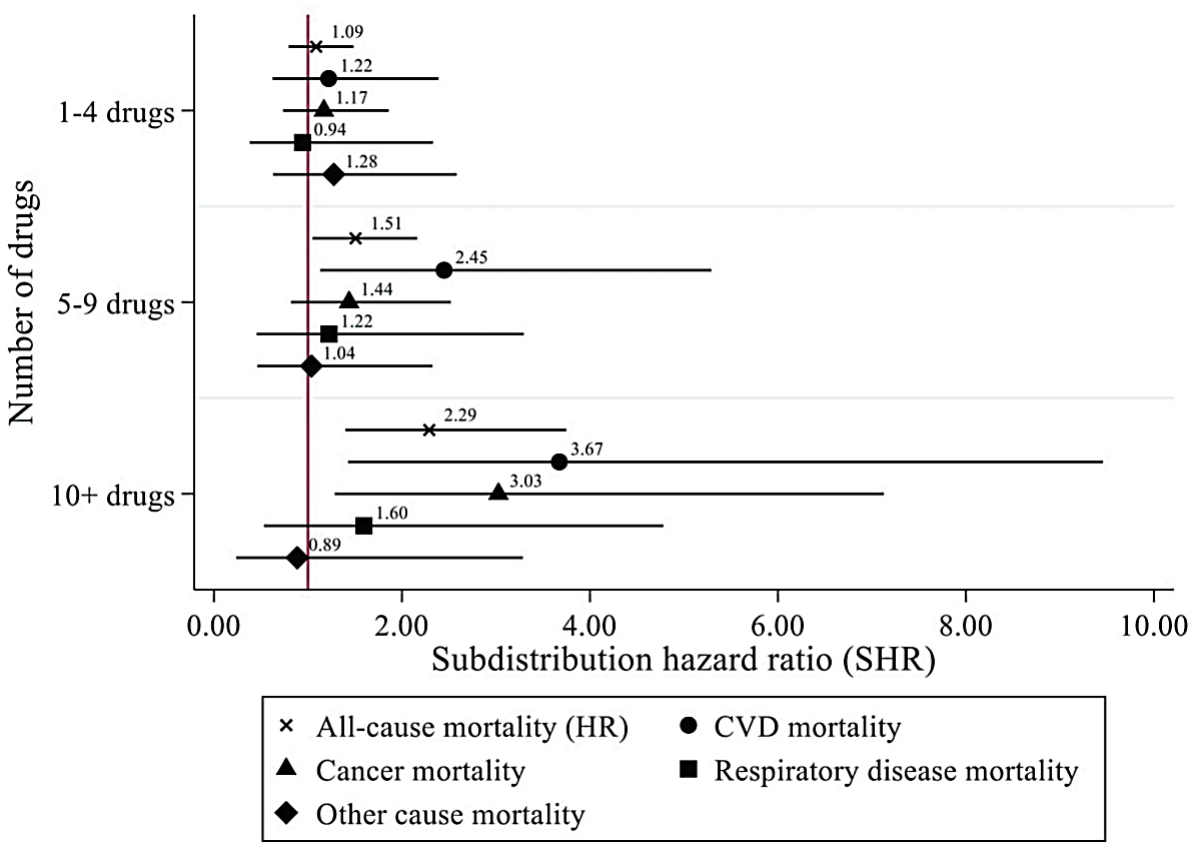
Associations between the number of concurrent drugs and mortality, England 2012–2018. CVD = cardiovascular disease; HR = hazard ratio.

**Figure 2.**
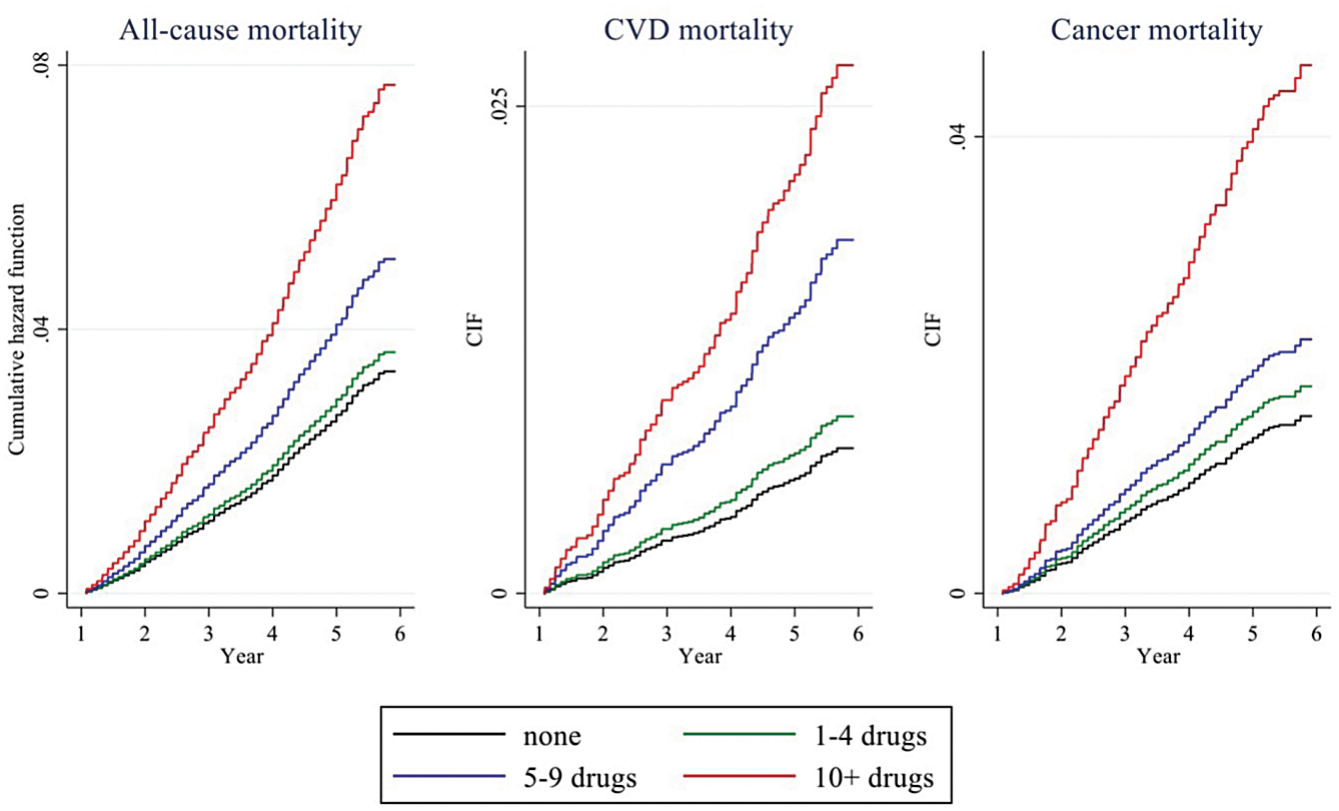
Polypharmacy performs differently in all-cause, CVD, and cancer mortality, England 2012–2018. CIF = cumulative incidence function; CVD = cardiovascular disease.

The results of cause-specific mortality analyzed by using competing-risks regression are presented in Figure 1. Polypharmacy was only related to a higher risk of CVD deaths (SHR = 2.45, 95% CI = 1.13, 5.29), while heightened polypharmacy was independently associated with CVD mortality (SHR = 3.67, 95% CI = 1.43, 9.46) and cancer mortality (SHR = 3.03, 95% CI = 1.29, 7.13). The 95% CIs of cause-specific mortality were much wider than all-cause mortality due to smaller sample sizes. The cumulative hazard function of all-cause mortality and cumulative incidence function of CVD and cancer mortality are displayed in Figure 2.

The results of sensitivity analyses are summarized in Supplementary Table S3. The first sensitivity analysis took known drug–disease interactions into account but showed no important differences from the primary analysis. Similarly, the second sensitivity analysis in which we included alcohol consumption with a reduced sample size because of missing data (*N* = 5 805), the dose–response relationship between polypharmacy and death was also observed. The third sensitivity analysis additionally involved people taking particular medications but without corresponding diagnoses (10.2%), but the estimates for polypharmacy and heightened polypharmacy remained quite robust. Furthermore, the adjustment for multimorbidity (defined as ≥2 long-term conditions) in sensitivity analysis 4 led to an increase in the HR associated with polypharmacy (HR = 1.86) and heightened polypharmacy (HR = 3.19) in comparison with the primary analysis (HR = 1.51 for polypharmacy; HR = 2.29 for heightened polypharmacy). However, there was a close relationship between polypharmacy and multimorbidity (Supplementary Table S4) so these estimates may be unreliable. Also, when we modeled all chronic conditions individually instead of combining some conditions into an illness count in a further sensitivity analysis, similar results were observed to the primary results. Finally, when we changed the reference group from none to 1–4 medications, the findings for all-cause mortality and causes of death were similar to the primary results, confirming the robustness of the findings (Supplementary Figure S1).

## Discussion

### Summary

Over a 6-year period, polypharmacy and heightened polypharmacy showed dose–response relationships with all-cause and CVD mortality among older adults in England. In addition, cancer mortality was associated with heightened polypharmacy. As expected, the present long-term conditions are a key factor in the association between polypharmacy and all-cause deaths, but the relationship with mortality was robust even after preexisting illness, and demographic and other factors were taken into account.

The robustness of the main findings was largely confirmed by sensitivity analyses, indicating that polypharmacy is an independent risk factor for death, including all-cause, CVD, and cancer deaths, among community-dwelling older adults. Multimorbidity appears to be an inappropriate assessment of health condition for older adults and to overestimate the risk of polypharmacy on deaths. This result justifies the main model and suggests that the risk of polypharmacy on death in our study is not over- or underestimated.

The underlying mechanism for the association between polypharmacy and mortality could be explained by 2 aspects: long-term conditions and regularly used medications. To some extent, the adjustment of long-term illness does not mean to take disease severity into account. Even the widely used Charlson Comorbidity Index only considers disease severity for particular illnesses (liver disease, diabetes, and solid tumor) (29). Take heart failure as an example, patients at the initial stage are likely to take fewer medications than those at advanced stages. Therefore, the number of medications can somehow represent disease severity, resulting in the association that polypharmacy performs as a predictor of death in older populations.

This association could be also attributed to medications and their potential interactions. Older people may have higher chances to develop problematic polypharmacy because of pharmacokinetic and pharmacodynamic alterations (30). For example, some medications become high-risk, or some drug–drug interactions become severe in older adults. Although major drug–drug interactions are expected to be avoided by general practitioners and pharmacists in clinical settings, minor drug–drug interactions could happen or may get worse in this population.

### Strengths and Limitations

This study has several strengths. Firstly, medication profiles were collected by nurses rather than self-reported and used to verify self-reported diagnoses. The verification and collection process help reduce misreporting bias. Secondly, we used a rigorous definition of polypharmacy that refers to medications in long-term use rather than temporary use of painkillers. Thirdly, OTC medications for chronic conditions were included, since some interactions between OTC and prescribed medications could be life-threatening, such as angiotensin-converting enzyme inhibitors in combination with potassium supplements (31). The study employed a nationally representative sample followed for up to 6 years for whom comprehensive characteristics, from sociodemographic characteristics to health status, were available. We adjusted statistically for a wide range of potential confounders than in previous research, including cognitive function, mobility impairment, lifestyle factors, and depressive symptoms. We also conducted competing-risks analyses for causes of death that should provide more accurate estimates as taking account of the event of interest and competing events simultaneously. Thus, the study provides strong evidence of associations between polypharmacy and deaths, accounting for characteristics not included in previous studies.

Some limitations of this study should also be acknowledged. Information on medication type, but not on duration, dose, and frequency, was collected during the nurse visit. Also, some combination medications were indistinguishable from a single medication, so the amount of polypharmacy may have been underestimated in these cases. The assessment was made at a single time point, and medications may have changed over the follow-up period.

### Comparison With Existing Literature

The association between polypharmacy and all-cause mortality observed in this study is supported by previous studies (10,21,32–36), while most of the studies that failed to find the association used logistic regression instead of time-to-event analysis (37–39). There are also variations in the literature as to which group is used as the reference category for polypharmacy, ranging from 0 to 1 medications to fewer than 10 medications (21,32–34). Our findings demonstrate that polypharmacy is related to higher risks of all-cause, CVD, and cancer deaths compared with either taking no medications or taking 1–4 medications. A systematic review (10) reported that the use of 1–4 medications was associated with death, but this was not found in our research. Many studies included in this review were based on nonrepresentative populations, for example, patients with heart failure or schizophrenia, had hospital-based or institutional-based study design, and had a short-term follow-up. These factors may account for differences from our results. In addition to long-term conditions, both disability and lifestyle factors somewhat attenuated the effect of polypharmacy on all-cause death, as has been observed in previous studies (21,34,40).

### Implications for Clinical Practice

The findings of this study imply that older adults with polypharmacy should be monitored carefully and given patient-centered health care such as medication review. Structured medication reviews have been recommended by the NICE (11), NHS Scotland (12), and NHS England (5) as clinical interventions for certain groups of people, for example, patients in care homes or people taking 10 or more medications. However, our findings suggest older adults who take 5–9 long-term medications are also at an increased risk of death. Besides, our results support the recommendation of NHS England that people with respiratory disease or CVD should be involved in the structured medication review (5) since these conditions were independently related to increased mortality. On top of that, diabetes patients may also need greater attention and proactive interventions. Further studies on polypharmacy are needed to provide more information on medication use within polypharmacy at a population level. Early intervention in medication use for community-dwelling older adults would ensure treatment appropriateness, reduce inappropriate or unnecessary medications, and potentially decrease polypharmacy-related adverse effects.

## Conclusion

Polypharmacy and heightened polypharmacy showed dose–response relationships with all-cause and CVD mortality among older adults in England over a 6-year follow-up period. Heightened polypharmacy was also related to a higher risk of cancer mortality. In addition to the structured medication reviews currently advised for heightened polypharmacy, our results emphasize that greater attention to polypharmacy in general for older people may be helpful in reducing adverse effects and improving older adults’ health.

## Supplementary Material

glab155_suppl_Supplementary_Data
