# Supplementary material for: Dose–Response Relationships Between Polypharmacy and All-Cause and Cause-Specific Mortality Among Older People
Source: J Gerontol A Biol Sci Med Sci. 2021 Jun 3;77(5):1002–8. doi: 10.1093/gerona/glab155 (PMC9071388; doi:10.1093/gerona/glab155)
Supplement: glab155_suppl_Supplementary_Data [file glab155_suppl_supplementary_data.docx]

**Supplementary data**

**Table S1 List of drug-disease interactions.**

|  | Drug | Disease |
| --- | --- | --- |
| 1 | Corticosteroids | Diabetes |
| 2 | Corticosteroids | Osteoporosis |
| 3 | Antipsychotics | Parkinson’s disease |
| 4 | Non-steroidal anti-inflammatory drugs (NSAIDs) | Hypertension |
| 5 | Anticholinergics/ Tricyclic antidepressants/ Benzodiazepines | Dementia |

**Table S2 Significant factors associated with all-cause mortality from the fully adjusted model, England 2012−2018.**

|  | HR (95% CIs) | *P* |
| --- | --- | --- |
| Number of concurrent drugs (Ref=none) |  |  |
| Polypharmacy (5−9 drugs) | 1.51 (1.05, 2.16) | **0.026** |
| Heightened polypharmacy (10+ drugs) | 2.29 (1.40, 3.75) | **0.001** |
| Age (years)^#^ | 1.11 (1.10, 1.12) | **<0.001** |
| Gender (Ref=men) | 0.60 (0.49, 0.72) | **<0.001** |
| Live with a partner (Ref=no) | 0.75 (0.61, 0.92) | **0.006** |
| Diabetes mellitus (Ref=no) | 1.28 (1.02, 1.60) | **0.035** |
| CHD (Ref=no) | 1.28 (1.02, 1.60) | **0.030** |
| Lung disease (including asthma) (Ref=no) | 1.28 (1.03, 1.60) | **0.028** |
| Obesity (Ref=normal BMI and waist circumference) |  |  |
| High BMI and waist circumference | 0.70 (0.55, 0.88) | **0.003** |
| Either high BMI or waist circumference | 0.76 (0.61, 0.95) | **0.015** |
| Current smoker (Ref=no) | 1.89 (1.44, 2.49) | **<0.001** |
| Low physical activity (Ref=moderate/high) | 1.54 (1.25, 1.89) | **<0.001** |
| Cognitive function^#^ | 0.95 (0.92, 0.97) | **<0.001** |

^#^ Per 1 unit increase

**Table S3 Sensitivity analyses of the associations between the number of concurrent drugs and all-cause mortality, England 2012−2018.**

|  | **None** | **1−4 drugs** | | **5−9 drugs**^*^ | | **10+ drugs**^*^ | |
| --- | --- | --- | --- | --- | --- | --- | --- |
| N=6295 (499 deaths) | HR | HR (95% CIs) | *P* | HR (95% CIs) | *P* | HR (95% CIs) | *P* |
| 1. Main model + drug-disease interactions | 1.00 (Ref) | 1.09 (0.79, 1.48) | 0.603 | 1.50 (1.04, 2.15) | **0.028** | 2.25 (1.37, 3.70) | **0.001** |
| 2. Main model + alcohol consumption^#^ | 1.00 (Ref) | 1.15 (0.82, 1.61) | 0.414 | 1.57 (1.06, 2.33) | **0.025** | 2.08 (1.19, 3.65) | **0.011** |
| 3. Main model + taking medications but without diagnoses^§^ | 1.00 (Ref) | 1.13 (0.82, 1.55) | 0.458 | 1.60 (1.10, 2.34) | **0.015** | 2.47 (1.48, 4.13) | **0.001** |
| 4. Main model with multimorbidity^†^ | 1.00 (Ref) | 1.19 (0.86, 1.65) | 0.299 | 1.86 (1.30, 2.67) | **0.001** | 3.19 (2.02, 5.06) | **<0.001** |
| 5. Main model with all chronic conditions^††^ | 1.00 (Ref) | 1.14 (0.83, 1.56) | 0.425 | 1.53 (1.06, 2.20) | **0.023** | 2.16 (1.31, 3.56) | **0.003** |

^*^ Polypharmacy refers to taking 5−9 drugs; heightened polypharmacy refers to taking 10 or more drugs

^#^ Reduced N=5805 (429 deaths)

^§^ A small proportion of people who took medications but did not report relevant diagnoses

^†^ Replace particular chronic conditions and the illness count with multimorbidity, defined as the coexistence of two or more chronic conditions

^††^ Replace the number of conditions with separate diagnoses, including hypertension, other heart problems, hyperlipidemia, arthritis, bone disease, psychiatric conditions, eye disease, gout or hyperuricemia, epilepsy, and inflammatory bowel disease

**Table S4 Number of concurrent drugs and multimorbidity.**

|  | None | 1−4 drugs | 5−9 drugs (polypharmacy) | 10+ drugs (heightened polypharmacy) |
| --- | --- | --- | --- | --- |
| Multimorbidity % (N) | 21.5 (396) | 71.6 (2211) | 96.9 (1176) | 98.7 (147) |

**Figure S1 Associations between the number of concurrent drugs and mortality with reference of 1−4 drugs, England 2012−2018.**
